# Case Report: Multimodal Imaging Guides the Management of an Eosinophilic Leukemia Patient With Eosinophilic Myocarditis and Intracardiac Thrombus

**DOI:** 10.3389/fcvm.2022.903323

**Published:** 2022-06-03

**Authors:** Jinping Si, Xinxin Zhang, Na Chen, Fangfang Sun, Ping Du, Zhiyong Li, Di Tian, Xiuli Sun, Guozhen Sun, Tao Cong, Xuemei Du, Ying Liu

**Affiliations:** ^1^Department of Cardiology, First Affiliated Hospital of Dalian Medical University, Dalian, China; ^2^Department of Nuclear Medicine, First Affiliated Hospital of Dalian Medical University, Dalian, China; ^3^Department of Radiology, First Affiliated Hospital of Dalian Medical University, Dalian, China; ^4^Department of Hematology, First Affiliated Hospital of Dalian Medical University, Dalian, China; ^5^Department of Ultrasonography, First Affiliated Hospital of Dalian Medical University, Dalian, China

**Keywords:** 18F-FAPI, eosinophilic leukemia, thrombotic, fibroblast activation protein, PET/CT

## Abstract

**Background:**

Eosinophilic leukemia (EL) is a rare, serious and potentially life-threatening condition characterized by the overproduction of eosinophils leading to tissue eosinophilic infiltration and damage. Although multiple organ systems may be involved, progressive eosinophilic myocarditis (EM) is the most common cause of morbidity and mortality. Early diagnosis and follow-up surveillance combined with multimodal imaging are crucial for appropriate treatment of EM.

**Case Summary:**

It’s a rare case of EL with EM and intracardiac thrombus in a 59-year-old patient who presented with asthenia for 3 weeks. Full blood count analysis indicated significant eosinophilia. Bone marrow aspirate revealed dysplastic eosinophilia and a FIP1L1-PDGFRA fusion gene (4q12) was detected, confirming EL. Echocardiography revealed EM with intracardiac thrombus. This was later confirmed by cardiac magnetic resonance imaging. The patient was commenced on imatinib and prednisolone and good clinical response was obtained. Through 18F-FAPI PET/CT imaging, we obtained *in vivo* visualization of fibroblast activation changes in the early stage of cardiac structure remodeling. With anti-fibrotic therapy after heart failure, the patient achieved a good clinical response.

**Conclusion:**

This case demonstrates *in vivo* visualization of fibroblast activation after EM. Multimodality imaging can provide early diagnosis and may guide tailored antifibrotic therapy in early stage of EM.

## Introductions

Eosinophilic myocarditis (EL) is a neoplastic condition with persistent eosinophilia as the main hematological abnormality and with the eosinophils being part of the neoplastic clone (1). These eosinophils have a clonal molecular genetic abnormality that excludes PDGFRA, PDGFRB, and FGFR1 rearrangements and PCM1-JAK2, ETV6-JAK2, and BCR-JAK2 fusion genes, especially a FIP1L1–PDGFRA fusion gene resulting from a cryptic deletion of part of the long arm of chromosome 4 (2). Reducing eosinophil levels both in peripheral blood and tissue, preventing end-organ damage and avoiding adverse thrombotic events are the three primary goals for the management of EL (3). EM, a common cardiac complication of EL and the major cause of morbidity and mortality which can occur in about 40–50% of patients (4), is an inflammatory disorder of the myocardium characterized by eosinophilic infiltration (5). EM usually undergoes acute necrotic, thrombotic, and fibrotic stages, which may overlap. Early diagnosis and follow-up surveillance combined with multimodal imaging are crucial for appropriate treatment of EM.

## Case Presentation

**FIGURE 1:**
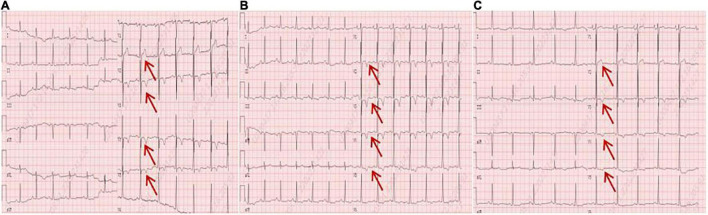
**(A)** An electrocardiogram on admission. **(B)** An electrocardiogram revealed a new ST segment depression with T-wave inversion in the precordial leads (arrows). **(C)** An electrocardiogram before discharge showed the normalization of ST segment and T wave (arrows).

**FIGURE 2:**
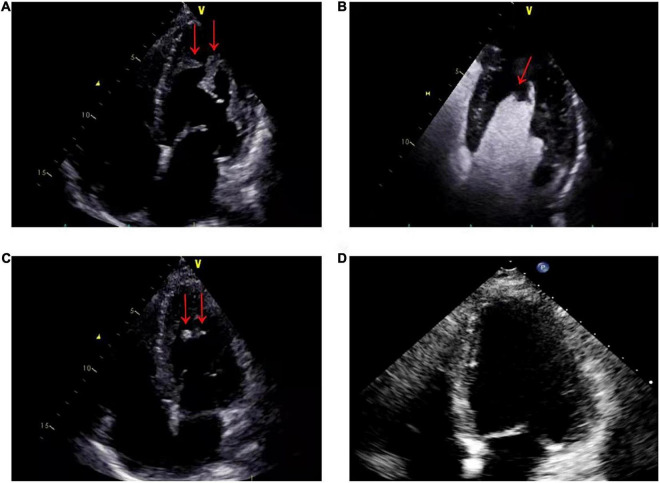
**(A)** On admission, echocardiography showed that the apex of the left ventricle was filled with a thrombotic mass measuring 3.9 cm × 2.2 cm (arrows). **(B)** On admission, echocardiographic view after venous injection of an ultrasound contrast agent: thrombus (arrow) at left ventricle apex. **(C)** Two months after therapy, echocardiography showed a marked reduction in the size of the mass, with a thrombotic mass measuring 6 mm × 13 mm (arrows). **(D)** Six months after therapy, echocardiography showed that left ventricular thrombus had resolved.

**FIGURE 3:**
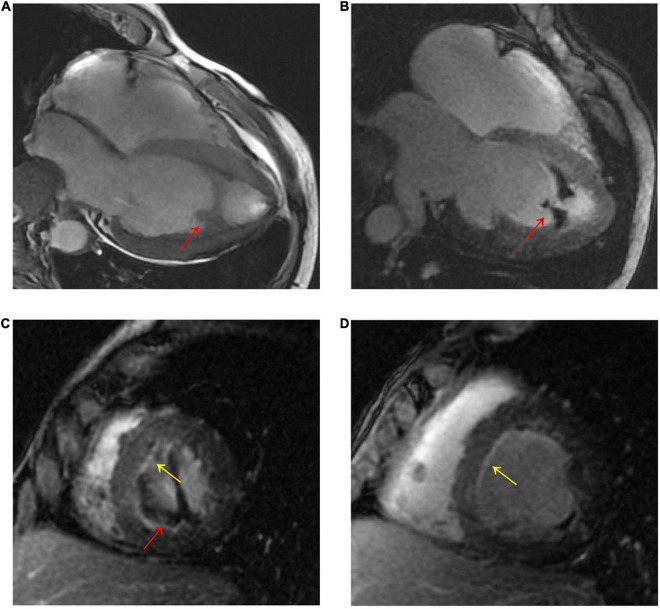
**(A)** CMR four-chamber cine sequence exhibited arc-like region in the LV apex (red arrow). **(B–D)**, Late gadolinium enhancement imaging showed LV apex arc-like unenhanced region (red arrow), and diffuse subendocardial enhancement in LV mid- to apical parts (yellow arrow). The patient was diagnosed with Loeffler’s endocarditis based on the typical clinical and imaging features.

A 56-year-old male presented with asthenia for 3 weeks. He had no food or drug allergies, no laboratory evidence of parasite, fungal or virus infection. Vital signs were normal with blood pressure of 130/80 mmHg, heart rate of 80 beats/min and respiratory rate of 12 breaths/min on presentation. Complete blood examination revealed hemoglobin 95 g/dL, total leukocyte count 27.74 × 10^9^/L with an absolute eosinophil count of 12.94 × 10^9^/L, accounting for 59.8% of cells, platelet count 55 × 10^9^/L, elevated N-terminal pro-brain natriuretic peptide (NT-proBNP) level (604.6 ng/L, normal range <450 ng/L) and C-reactive protein (11 mg/L, reference: <8 mg/L). High Sensitivity Cardiac Troponin I (hs-cTnI) level was within the normal range. Other laboratory results were unremarkable, including anti-nuclear antibody screen. The electrocardiogram (ECG) revealed sinus rhythm (Figure 1A). Neither coronary computed tomographic angiography nor encephalic computed tomography showed significant lesions. Echocardiography demonstrated slightly increased chamber size, normal left ventricular systolic function (left ventricular ejection fraction of 57%), and abnormal diastolic function of grade III. There was a large, heterogeneous cardiac mass (39 mm × 22 mm, arrow) in the left ventricle (LV). The most probable diagnosis was Löffler’s endocarditis (LE; cardiac involvement in hypereosinophilia) with intracardiac thrombus (Figures 2A,B). A cardiac magnetic resonance (CMR) short-axis image showed a curved line of late gadolinium enhancement (LGE) located subendocardial in the apical-middle heart of LV. The arc-shaped unenhanced area in the left ventricular cavity and the medical history were consistent with eosinophilic endocarditis with subendocardial thrombosis (3) (Figure 3). Because of the drastically increased eosinophils, we suspected a myeloproliferative disorder. We did a bone marrow examinations including aspiration cytology, biopsy, cytogenetic, and gene rearrangement analysis. While waiting for the results of the examinations, on day 3 of his admission, the patient complained of progressive development of chest pain. The electrocardiographic examination showed a new ST segment depression with T-wave inversion in precordial leads (Figure 1B). Laboratory findings demonstrated an elevated hs-cTnI and NT-proBNP of 2.72 and 1,789 ng/L, respectively, indicating ongoing myocardial damage. After treatment with nitrates to dilate the coronary arteries, the chest pain disappeared. As the symptoms were related to EM, the cause might be coronary artery spasm or a small thromboembolism obstructing the microcirculation, but the patient refused to undergo coronary angiography. Subsequently, bone marrow aspirate revealed dysplastic eosinophilia and eosinophilic promyelocytes. A normal karyotype (46, XY) was present and a FIP1L1-PDGFRA fusion gene (4q12) was detected. The marrow B- and T-cell receptor rearrangement analysis and flow cytometry showed no B- or T-cell clone, confirming EL. The patient was treated with glucocorticoid. The initial medication prescribed was intravenous methylprednisolone (60 mg per day for 3 days and then 40 mg per day for 4 days), then which was switched to oral prednisolone (50 mg per day), with a gradual tapering of the doses and imatinib mesylate (a tyrosine kinase inhibitor) of 100 mg/day was commenced. After only 4 days on imatinib and prednisolone, and the patient’s eosinophil cell levels returned to normal (the lowest level was 0.01 × 10^9^/L). After diagnosis of intracardiac thrombus, he was initially treated with low-molecular-weight heparin (1 mg/kg/dose q12 h) for 7 days. The heparin was gradually switched to warfarin. Warfarin was adjusted to a PT INR of 2–3. Before discharge, the hs-cTnI and NT-proBNP levels had significantly decreased to 0.032 and 617.8 ng/L, respectively. Furthermore, an ECG showed the normalization of ST segment and T wave (Figure 1C).

**FIGURE 4:**
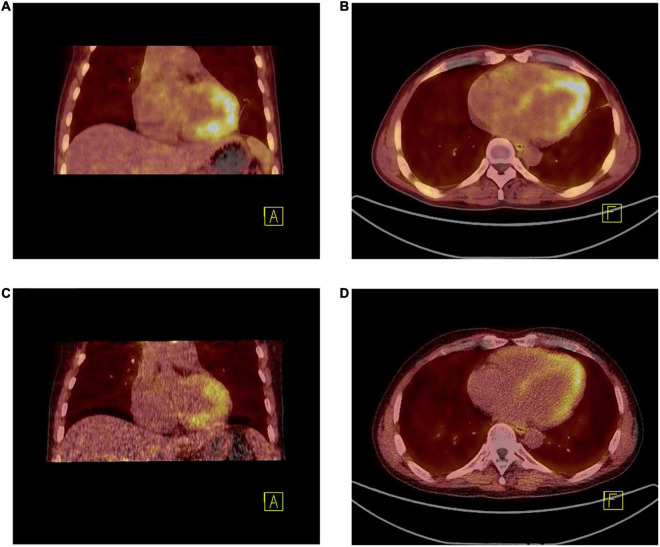
**(A,B)** 18F-FAPI PET/CT fused image demonstrated the uptake [10.10 (SUVmax), 8.75 (SUVpeak), and 1.92 (SUVmean)] was much higher than that after 2 months of treatment **(C,D)** [8.40 (SUVmax), 6.91 (SUVpeak), and 1.32 (SUVmean)].

The patient underwent twice 18F-FAPI PET/CT for the visualization of cardiac fibroblast activation during treatment. Before discharge, on whole-body 18F-FAPI PET imaging, heterogeneously increased accumulations of 18F-FAPI were seen in endomyocardial. No other suspected fibrosis with 18F-FAPI accumulation was found (Figures 4A,B). Given the presence of myocardial fibrosis, he was treated for myocardial fibrosis and heart failure with angiotensin receptor-neprilysin inhibitor (ARNI), β-blockers and spironolactone. Good clinical response was obtained and after 2 months of treatment, follow-up PET/CT revealed that accumulations of 18F-FAPI in endomyocardial were significantly reduced (Figures 4C,D).

The patient was closely followed up in both cardiology and hematology outpatient clinics over a course of 2 months. The eosinophil counts remained within the normal range, when the dose of prednisolone was gradually tapered to 5 mg per day. Peripheral blood examination gave the following results: hemoglobin, 124 g/dL; total leukocyte count 8.66 × 10^9^/L with 6.71% eosinophils; and platelet count, 177 × 10^9^/L. While remaining on warfarin, imatinib mesylate, prednisolone and heart failure therapy, he had no specific symptoms on evaluation in the outpatient clinic. Follow-up echocardiogram revealed normal left ventricular size and rapid regression of the left ventricular mass (6 mm × 13 mm, arrow) (Figure 2C).

During a 6-month follow-up, there was no recurrence of hypereosinophilia. The disease remained clinically stable. Echocardiogram showed that left ventricular thrombus had resolved (Figure 2D).

## Discussion

The diagnostic features of EL include (1) persistent proliferation of eosinophil precursors and (2) marked eosinophilia in peripheral blood (eosinophils >1.5 × 10^9^/L) and bone marrow. Eosinophils play a role in host defense and immune regulation by secreting intracytoplasmic granules containing cytotoxic molecules, cytokines and chemokines, which can lead to end-organ injury. EM is a common cardiac complication of hypereosinophilia. The pathophysiology of EM progresses through three stages, namely acute necrotic stage, thrombotic stage and fibrotic stage, which may overlap (6). The prognosis for EM is often poor, with a 5-year mortality rate of up to 50% (7). Besides, endomyocardial exhibits various cardiac manifestations depending on the severity and extent of the endomyocardial damage caused by eosinophilic infiltration (8). Therefore, EM often leads to the development of heart failure. Treatment of EM to prevent further deterioration includes halting of eosinophilic infiltration, counteraction of inflammation reaction, and prevention of myocardial fibrosis/intracardiac thrombus formation, in addition to symptomatic treatment of heart failure. If left untreated, serious complications such as valvular regurgitation or restrictive cardiomyopathy may occur at an advanced stage of EM. However, early and proper diagnosis is particularly challenging due to the subclinical presentation and the lack of characteristic signs in early stages of disease. In our case, the EM was most likely in the second or early third stage due to the presence of LV thrombus and fibroblast activation; echocardiography and PET-CT follow-up revealed improved LV function and suppressed fibroblast activation suggesting that the prompt treatment was protective against permanent cardiac dysfunction.

In this report, we describe a case of EL with EM and LV thrombus. Our case may offer several valuable clinical lessons. Imatinib therapy is indicated in all patients with either a chronic myeloid leukemia or a myeloproliferative disorder with PDGFRB and PDGFRA abnormalities (9). Corticosteroids are used as a cornerstone of myocarditis treatment, but its mechanism of action is not fully understood. It is thought that corticosteroids effectively inhibit the proliferative, developmental maturation pathway of eosinophils, thereby promoting redistribution of peripheral blood eosinophils (10). So a very effective treatment with imatinib and corticosteroids was possible in this case with FIP1L1–PDGFRA fusion gene, and early treatment with imatinib and corticosteroids did show favorable results. In the case described, the patient developed chest pain during treatment, elevated leukocytosis, eosinophilia, hs-cTnI, and NT-proBNP were evident and this was explained by infiltration of eosinophils into the myocardium. This infiltration leads to mitochondrial dysfunction, myocardial cell damage and necrosis through the release of toxic granules, cationic proteins, pro-inflammatory cytokines and oxygen free radicals. Additionally, eosinophil infiltration can lead to coronary vasculitis and the release of cytokines that might cause coronary artery spasm (11). Therefore, in our case with chest pain, pronounced eosinophilia levels, and normal coronary computed tomography angiography, the diagnosis of EM should be considered. In some individuals, EM masquerades as acute coronary syndrome, with severe chest pain, electrocardiographic changes, and elevated serum levels of troponins (12). If patients who have hypereosinophilia presenting as acute coronary syndrome only receive stent implantation and anti-angina pectoris, coronary spasm will occur repeatedly and may even lead to sudden death. However, if the glucocorticoid or immunosuppressor is administered their condition will be improved (13, 14). In addition to first-line therapy (corticosteroids), early and aggressive anticoagulation therapy shall be ensured (15). While there is no standard recommendation for the use of anticoagulation for EL in the presence of thrombosis, anticoagulation should be considered in patients at high risk (i.e., with evidence of intracardiac thrombosis, deep venous thrombosis or recurrent thromboembolic events) with a view to treating this potentially fatal complication (16). In our case, after anticoagulation treatment, the patient achieved good therapeutic response.

CMR allows the visualization of myocardial inflammation, mural thrombus and fibrosis, with high sensitivity, specificity and accuracy, and the differentiation of inflammation from fibrosis based on the enhancement on LGE imaging (8, 17). EM is often associated with diffuse or patchy subendocardial LGE, which was shown in our patient. However, CMR is not specific for assessing early cardiac structural remodeling.

The newly developed PET tracer FAPI specifically targets fibroblast activation protein, which allows *in vivo* visualization of fibroblast activation during an early stage of cardiac structural remodeling (18). Currently, there are no other imaging studies of human fibroblast activation using this novel biosignal for EM. Given the limited treatment approach in an irreversible stage, earlier diagnosis and therapeutic intervention in EM progression is essential. Although endomyocardial biopsy (EMB) is the cornerstone of EM histological diagnosis, it is an invasive procedure and inflammatory cell infiltration is heterogeneous distributed, resulting in low diagnostic accuracy (19). Additionally, EMB is not indicated in all patients with suspected EM (5). In our case, because the patient remained in good condition after starting corticosteroid therapy, biopsy was not performed, and the presence of left ventricular thrombosis was also an influencing factor. PET/CT imaging can safely provide valuable information about the location, extent, and pattern of endomyocardial fibrosis. Moreover, excessive fibrosis development is suggested to cause a progressive decline in ventricular diastolic and systolic function, which may lead to the development of chronic heart failure (20). Our case illustrates how EM due to EL could cause endomyocardial fibrosis, diastolic dysfunction, and thrombus (21). The FAPI uptake peaks on day 6 following ischemia (22), in the present case, the patient underwent FAPI imaging 16 days after chest pain, and the FAPI uptake area also did not conform to the patterns of myocardial ischemia. Thus, we excluded myocardial ischemia and considered that the FAPI imaging changes were caused by EM. Affected myocardium showed a partial to complete match between tracer uptake and confirmed lesion by contrast echocardiography and CMR. Pathologic features of EM fibrosis were identified. As fibroblast activation protein expression is low in most normal organs, it provides an interesting target for PET imaging to assess the severity of fibrosis (23). 18F-FAPI may represents a highly promising, novel biosignal for monitoring short-term structural remodeling processes and guiding tailored antifibrotic therapy in hypereosinophilia (24, 25). In our case, 18F-FAPI PET/CT, CMR and Echocardiography together present synergies that go beyond the limitations that each examination presents separately.

In conclusion, this case illustrates how EM due to EL could cause endomyocardial fibrosis, diastolic dysfunction, and thrombus. Advances in diagnostic imaging techniques may aid in the diagnosis of EM. Multimodality imaging can provide early diagnosis and potentially guide tailored antifibrotic therapy in EM.

## Data Availability Statement

The original contributions presented in the study are included in the article/supplementary material, further inquiries can be directed to the corresponding authors.

## Ethics Statement

The studies involving human participants were reviewed and approved by the First Affiliated Hospital of Dalian Medical University. The patients/participants provided their written informed consent to participate in this study. Written informed consent was obtained from the individual(s) for the publication of any potentially identifiable images or data included in this article. Written informed consent was obtained from the participant for the publication of this case report.

## Author Contributions

YL, XD, and TC contributed to the conception of the case report. JS and XZ contributed to the management of the patient and manuscript writing. NC contributed to the manuscript preparation and clinical data collection. FS, ZL, and DT contributed to the imageological examination. PD, GS, and XS contributed to the eosinophilic leukemia, eosinophilic myocarditis, and intracardiac thrombus treatment of the patient. All authors contributed to the article and approved the submitted version.

## Conflict of Interest

The authors declare that the research was conducted in the absence of any commercial or financial relationships that could be construed as a potential conflict of interest.

## Publisher’s Note

All claims expressed in this article are solely those of the authors and do not necessarily represent those of their affiliated organizations, or those of the publisher, the editors and the reviewers. Any product that may be evaluated in this article, or claim that may be made by its manufacturer, is not guaranteed or endorsed by the publisher.
